# Effects of Vaccination with 10-Valent Pneumococcal Non-Typeable *Haemophilus influenza* Protein D Conjugate Vaccine (PHiD-CV) on the Nasopharyngeal Microbiome of Kenyan Toddlers

**DOI:** 10.1371/journal.pone.0128064

**Published:** 2015-06-17

**Authors:** Leah M. Feazel, Stephanie A. Santorico, Charles E. Robertson, Mahfudh Bashraheil, J. Anthony G. Scott, Daniel N. Frank, Laura L. Hammitt

**Affiliations:** 1 Division of Infectious Diseases, University of Colorado School of Medicine, Aurora, CO, United States of America; 2 Dept. of Mathematical & Statistical Sciences, University of Colorado Denver, Aurora, CO, United States of America; 3 Dept. of Molecular, Cellular, and Developmental Biology, University of Colorado Boulder, CO, United States of America; 4 KEMRI-Wellcome Trust Research Programme, Centre for Geographic Medicine-Coast, Kilifi, Kenya; 5 Nuffield Department of Clinical Medicine, University of Oxford, Oxford, United Kingdom; 6 London School of Tropical Medicine and Hygiene, London, United Kingdom; 7 University of Colorado Microbiome Research Consortium (MiRC), Aurora, CO, United States of America; 8 Department of International Health, Johns Hopkins Bloomberg School of Public Health, Baltimore, MD, United States of America; Public Health England, UNITED KINGDOM

## Abstract

**Objective:**

Pneumococcal conjugate vaccines reduce the prevalence of vaccine serotypes carried in the nasopharynx. Because this could alter carriage of other potential pathogens, we assessed the nasopharyngeal microbiome of children who had been vaccinated with 10-valent pneumococcal non-typeable *Haemophilus influenzae* protein-D conjugate vaccine (PHiD-CV).

**Methods:**

Profiles of the nasopharyngeal microbiota of 60 children aged 12-59 months, who had been randomized to receive 2 doses of PHiD-CV (n=30) or Hepatitis A vaccine (n=30) 60 days apart, were constructed by 16S rRNA gene pyrosequencing of swab specimens collected before vaccination and 180 days after dose 1.

**Results:**

Prior to vaccination, *Moraxella catarrhalis* (median of 12.3% of sequences/subject), *Streptococcus pneumoniae* (4.4%) and *Corynebacterium spp*. (5.6%) were the most abundant nasopharyngeal bacterial species. Vaccination with PHiD-CV did not significantly alter the species composition, abundance, or prevalence of known pathogens. Distinct microbiomes were identified based on the abundances of *Streptococcus*, *Moraxella*, and *Haemophilus* species. These microbiomes shifted in composition over the study period and were independent of age, sex, school attendance, antibiotic exposure, and vaccination.

**Conclusions:**

Vaccination of children with two doses of PHiD-CV did not significantly alter the nasopharyngeal microbiome. This suggests limited replacement carriage with pathogens other than non-vaccine strains of *S*. *pneumoniae*.

**Trial Registration:**

clinicaltrials.gov NCT01028326

## Introduction

Pneumococcal disease is estimated to cause illness in 13.8 million children under 5 years of age, with >800,000 deaths annually.[1] In countries where pneumococcal conjugate vaccine (PCV) has been introduced into the childhood immunization schedule, it has substantially reduced the incidence of vaccine-type invasive pneumococcal disease (IPD) among young children.[2] In addition to generating a serum antibody response, vaccination with conjugate PHiD-CV also results in a reduction in the prevalence of nasopharyngeal carriage of vaccine-type pneumococci.[3] The incidence of vaccine-type IPD among unvaccinated children and adults have also been reduced through decreased transmission of pneumococcal infection from younger, vaccinated children. [4]

Vaccine-induced changes in nasopharyngeal pneumococcal carriage may have other effects on the nasopharyngeal microbiome, including increased carriage of pathogenic species not targeted by vaccination.[5] To examine the effects of pneumococcal vaccination on nasopharyngeal microbial communities, we applied culture-independent molecular techniques of bacterial identification, in parallel with traditional culture techniques, to nasopharyngeal specimens collected from children before and after vaccination with the 10-valent pneumococcal conjugate vaccine (10-valent pneumococcal non-typeable *Haemophilus influenzae* protein D conjugate vaccine [PHiD-CV]; Synflorix).

## Methods

### Study design and subjects

This study was nested within a double-blind randomized controlled trial involving children aged 12–59 months residing in Malindi District, Kenya, a rural community on the Kenyan coast (NCT01028326).[6] Subjects eligible for this sub-study had been randomized to receive vaccines on days 0, 60 and 180 in either of two vaccination sequences: 1) PHiD-CV, PHiD-CV, diphtheria/tetanus/acellular pertussis vaccine (DTaP) (n = 200) or 2) Hepatitis A vaccine (HAV), DTaP, and HAV (n = 200; control group). A random sample of 30 subjects from each of these two groups was selected for microbiome analysis by assigning a random number to the samples in each group (RAND() function in Microsoft Excel) and selecting the lowest 30 numbers. The Kenya National Ethical Review Committee (SSC 1635), the Oxford Tropical Ethical Review Committee (OxTREC 54–09), and the Colorado Multiple Institutional Review Board (protocol 11–0352) approved the study. Parents/guardians of participants provided written informed consent.

### Sample collection

Nasopharyngeal specimens were collected from each subject (one swab from each side of the nose) on days 0 and 180 using a rayon swab (Medical Wire Co, UK). Specimens were collected by passing the swab through the nostril, along the floor of the nasal cavity until it touched the posterior nasopharyngeal wall, where it was left for 2–3 seconds, rotated, and removed. One swab was placed in a sterile 2mL microcentrifuge tube containing skim milk, tryptone, glucose and glycerin (STGG) medium and processed at the KEMRI-Wellcome Trust Programme laboratory in Kilifi, Kenya in accordance with WHO recommendations.[7] Isolates of *S*. *pneumoniae* were identified from gentamicin-blood agar by Optochin susceptibility testing. Isolates of *H*. *influenzae* were identified from bacitracin-chocolate agar by X and V factor dependence. The second nasopharyngeal swab was placed in a sterile 2 mL microcentrifuge tube containing ethanol and stored in a freezer at -80°C prior to microbiome analysis.

### Microbiome analyses

Microbiome analysis was conducted on nasopharyngeal swab specimens collected at day 0 and day 180, as well as negative control swabs placed in ethanol at the study site and extracted/analyzed in the same manner as the specimens. Bacterial profiles were determined by broad-range PCR of 16S rRNA genes and phylogenetic sequence analysis. DNA was extracted using the UltraClean fecal DNA kit (MoBio, Inc). Amplicons of the 16S rRNA gene (~500 base pairs; primers 27FYM+3 and 534R)[8, 9] were generated via broad-range PCR (30–36 cycles) using 5’-barcoded reverse primers.[10] PCR yields were normalized using a SequalPrep kit (Invitrogen, Carlsbad, CA), pooled, lyophilized, and gel purified, as previously described.[11] Pyrosequencing of pooled amplicons was conducted by the Center for Applied Genomics at the University of Toronto on a 454/Roche Life Sciences GS-FLX instrument using Titanium chemistry (Roche Life Sciences, Indianapolis, IN).

Pyrosequences were sorted into libraries by barcode and filtered for quality using *bartab* software.[10] Bases at 5’ and 3’ ends with mean quality scores <20 over a 10 nucleotide window were deleted. Sequences with less than 200 nucleotides or more than one ambiguous nucleotide were discarded. The mean trimmed sequence length was ~340 base pairs. The *Infernal* RNA alignment tool[12] was used to screen all sequences in terms of their fidelity to a Covariance Model (CM) derived from 16S rRNA secondary structure models provided by the lab of Dr. Robin Gutell.[13] Sequences that did not match a bacterial CM model were removed from all subsequent analyses. Chimera screening was performed using the software *ChimeraSlayer*, which requires that sequences be previously aligned with the software *NAST-iEr*.[14] Putative chimeras and other sequences that could not be aligned by *NAST-iEr* were removed from subsequent analyses.

Genus-level taxonomic classifications were produced by the *RDP Classifier* software, which performs naïve Bayesian taxonomic classification versus the RDP training set.[15] Species-level taxonomic precision was obtained via BLAST[16] against a database of sequences obtained from the Silva database (version 104) that were annotated as isolates.[17] Species-level classifications were assigned when a nasopharyngeal swab sample sequence overlapped the database sequence by at least 95% with at least 99% sequence identity and the Silva database-derived taxonomy matched the RDP classifier genus level classification. Pyrosequences were clustered into operational taxonomic units (OTUs) on the basis of these taxonomic assignments. The prevalence of an OTU represented the percentage of subjects in a group in which the OTU was detected, whereas the relative abundance was calculated as the number of sequences belonging to a particular OTU divided by the total sequence count for a subject. Pyrosequences were submitted to the NCBI Short Read Archive under BioProject PRJNA229922. Because 16S rRNA sequences do not differentiate between *S*. *pneumoniae* strains, serotype replacement is not detectable using this methodology.

Ecological indices[18] of richness (S_obs_, S_chao_), diversity (Shannon’s diversity [H_o_], evenness [H_o_/H_max_]), and coverage (Good’s index) were computed with the software tool *biodiv.[19]* These indices were estimated through bootstrap resampling and rarefaction of the OTU distributions obtained from each specimen. All 16S amplicon libraries were sequenced to >95% coverage.

### Statistical analyses

The R-statistical package (v.2.14.0) was used for all statistical analyses.[20] The vaccine and control group demographics were compared using Wilcoxon rank sum tests for continuous variables and Fisher exact tests for categorical outcomes. Exact confidence intervals for odds ratios were constructed based on Fisher’s exact test. The change in abundance of an OTU through time was assessed by first subtracting the baseline percent abundance from the abundance measured at 180 days following vaccination; these data were then analyzed using a t-test with significance assessed through 1,000,000 permutations for testing no change within each treatment arm and for comparing equal change between treatment arms. The heatmap in Fig 2 was constructed in R using the heatmap.2 function, with hierarchical clustering based on Euclidean distances (*dist* function) and complete linkage (*hclust* function). Differences in OTU abundance between the nasopharyngeal types defined by this hierarchical clustering were analyzed using a two-part statistic,[21] which combines the results of a test of proportions with a Wilcoxon rank test. All tests of null hypotheses were evaluated at α = 0.05.

## Results

Among the 60 specimens selected, PCR amplification of the 16S rRNA gene failed in one or both swabs in six subjects (five subjects in the PHiD-CV group and one in the control group). These were excluded from further analysis. None of the negative control swabs yielded a discernible PCR product. Participant characteristics were similar between vaccine groups (Table 1). Microbes detected by broad-range PCR amplification and pyrosequencing of bacterial 16S rRNA genes of nasopharyngeal swabs collected on day 0 and 180-days post-vaccination are presented in Table 2. A median of 3,114 (interquartile range [IQR] = 1,399–8,547) high-quality pyrosequencing reads were obtained per specimen. The median Good’s coverage score, a measure of completeness of sequencing, was 99.6% (IQR = 98–100%), indicating that the depth of sequencing was sufficient to fully describe the biodiversity of specimens. No significant differences were observed in either the number of pyrosequences or depth of coverage between treatment groups, as assessed by t-test (p = 0.35 and 0.96 respectively; Table 2).

**Table 1:** Characteristics of participants

Characteristics	PHiD-CV group N = 25	Control group N = 29	p-value
**Age in months, mean (standard deviation)**	31 (15)	31 (16)	0.70
**Female, n (%)**	13 (52)	17 (59)	0.78
**Cigarette exposed, n (%)**	10 (40)	6 (21)	0.15
**Number of children in household, mean**	2.9	3.0	0.35

**Table 2:** Relative abundance of common nasopharyngeal bacterial 16S rRNA sequence types

Taxa	All Subjects	PHiD-CV Group (N = 25)^a^		Control Group (N = 29)^a^		Day 180-Day0
	Day 0	Day 0	Day 180	Day 0	Day 180	Comparison (p-value)^b^
**Proteobacteria**	**56.9% (33.7–70.6)**	**58.6% (31.4–70.2)**	**61.7% (46.2–78.3)**	**53.8% (36.1–70.6)**	**57.1% (43.6–69.8)**	0.74
*Haemophilus influenzae*	*1*.*6% (0–9*.*8)*	*1*.*6% (0–7*.*9)*	*1*.*0% (0–4*.*9)*	*2*.*0% (0–13*.*8)*	*2*.*5% (0–12*.*6)*	0.85
*Moraxella catarrhalis*	*12*.*3% (3*.*7–24*.*5)*	*15*.*7% (3*.*4–28)*	*12% (1–24*.*6)*	*9*.*2% (3*.*7–18*.*8)*	*4*.*2% (1*.*4–13*.*1)*	0.65
*Moraxella nonliquefaciens*	*2*.*1% (0*.*6–10)*	*2*.*5% (1*.*2–9*.*5)*	*4*.*0% (0*.*8–14)*	*1*.*4% (0*.*3–10*.*2)*	*2*.*4% (0*.*1–8*.*9)*	0.47
**Firmicutes**	**25.9% (15–46.8)**	**20.1% (11.8–44.8)**	**18.2% (8.6–46.6)**	**26.6% (19.9–46.9)**	**31.6% (15.6–41.2)**	0.66
*Streptococcus pneumoniae*	*4*.*4% (0*.*2–25*.*4)*	*4*.*0% (0*.*3–32*.*3)*	*10*.*3% (0*.*4–37*.*7)*	*4*.*9% (0–21*.*1)*	*10% (0*.*9–35*.*3)*	0.67
**Actinobacteria**	**7.8% (1.8–21.6)**	**8.5% (1.5–15.8)**	**5.1% (0.9–9.2)**	**6.9% (2.3–22.1)**	**2.1% (0.5–15.2)**	0.18
*Corynebacterium spp*.	*5*.*6% (1*.*7–19*.*8)*	*8*.*5% (0*.*9–15*.*4)*	*3*.*8% (0*.*8–7*.*7)*	*5*.*2% (2–21*.*1)*	*2*.*1% (0*.*4–15*.*1)*	0.45
**Bacteroidetes**	**0.4% (0.1–3.8)**	**0.7% (0.2–4.1)**	**1.0% (0–4.2)**	**0.3% (0.1–2.4)**	**0.3% (0–3.3)**	0.92
**Other Phyla**	**0% (0–0.2)**	**0% (0–0.3)**	**0.1% (0–0.2)**	**0.1% (0–0.2)**	**0% (0–0.2)**	0.15
**Sequences per specimen**	3730	2104	2605	4939	2678	0.35
(Interquartile range)	(1649–9799)	(1146–9587)	(1610–7393)	(2231–10112)	(856–7046)	
**Good’s Coverage**	99.58%	99.60%	99.70%	99.60%	99.80%	0.96
**Shannon Diversity**	2.91	2.71	2.75	2.92	2.46^**c**^	0.29

^a^ Median relative abundances of sequences classified for most abundant phyla and selected species.

^b^ PHiD-CV group vs control group; p-values for comparison of the change in relative abundances over time are from the results of the multiple permutation t-test; p-values for “Sequences per Specimen”, “Good’s Coverage”, and “Shannon Diversity” are from 2-tailed t-test.

^c^ P = 0.02 for Day 180 vs Day 0 comparison of control group. P = 0.85 for PHiD-CV comparison of Day 180 vs Day0.

**Fig 1:**
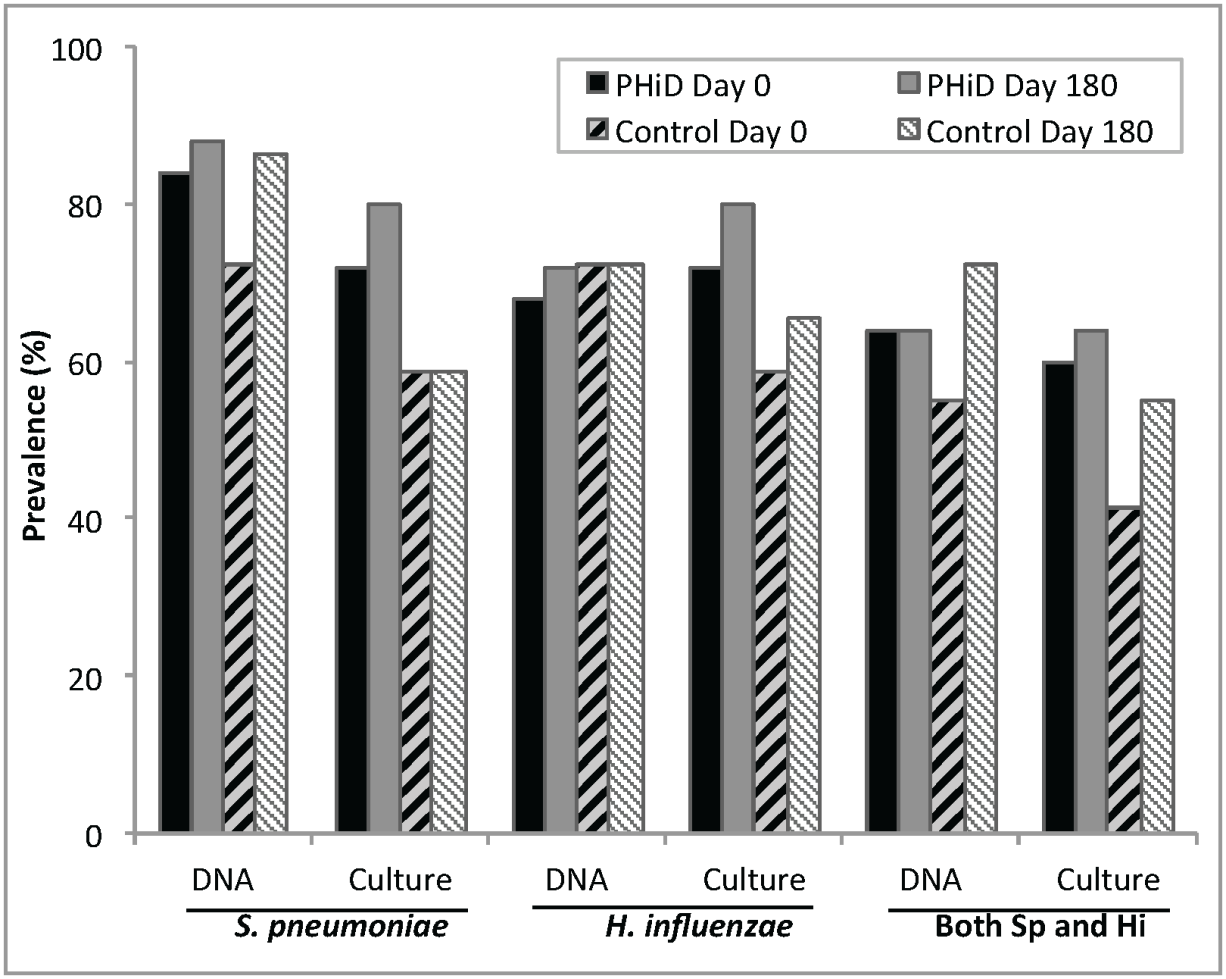
Prevalence of nasopharyngeal *S*. *pneumoniae* and *H*. *influenzae*. Prevalence is expressed as percentage of subjects positive for a bacterial species as measured by either 16S rRNA gene pyrosequencing or bacterial culture. PHiD-CV: 10-valent pneumococcal non-typeable *Haemophilus influenzae* protein-D conjugate vaccine treatment group. Control: Hepatitis A vaccine treatment group. Sp: *S*. *pneumoniae*. Hi: *H*. *influenzae*.

The change in relative abundance of OTUs over time did not significantly differ between vaccinated and control group participants, as assessed by 16S rRNA pyrosequencing (Fig 1, Table 2). Prior to vaccination, the most abundant bacterial species-level OTUs detected in the nasopharynx of all study participants (Table 2) were those of *Moraxella catarrhalis* (12.3% median relative abundance of pre-vaccination 16S rRNA sequences), *M*. *nonliquefaciens* (2.1%), *S*. *pneumoniae* (4.4%), and *H*. *influenzae* (1.6%). Corynebacterial species comprised an additional 5.6% median relative abundance among the pre-vaccination specimens. Staphylococci, including *Staphylococcus aureus*, were rare in the dataset, comprising <0.03% of total sequences. At baseline, the prevalence, as detected by pyrosequencing and bacterial culture, was 78% (42/54) and 65% (35/54), respectively, for *S*. *pneumoniae* and 70% (38/54) and 65% (35/54) for *H*. *influenzae* (Fig 1). *M*. *catarrhalis* was detected by pyrosequencing in 96% (52/54) of subjects at baseline (not assessed by culture). Neither the relative abundances nor the prevalences of these three potential pathogens changed significantly following vaccination with either PHiD-CV or control HAV (Fig 1, Table 2). Although the control group experienced a greater increase in *S*. *pneumoniae* prevalence than did the PHiD-CV arm, the difference was not statistically significant (14% vs. 4% increase, p = 0.36; Fig 1).

We observed co-carriage at baseline by *S*. *pneumoniae* and *H*. *influenza*e in 59% (32/54) of subjects by pyrosequencing and 50% (27/54) by culture (S1 Table). The odds ratio for detection of *S*. *pneumoniae* given the presence of *H*. *influenzae* was 3.2 (95% CI: 0.7–14.8; *p* = 0.15) by pyrosequencing and 4.5 (95% CI: 1.2–18.4; *p* = 0.02) by culture. In contrast, the prevalence of *M*. *catarrhalis* was not associated with that of *S*. *pneumoniae* (*p* = 0.40) or *H*. *influenzae* (*p* = 0.51).

A median of 22 genera (IQR = 13–33) were detected per child at baseline; however, the majority of DNA sequences (~80%) belonged to only four genera: *Moraxella* (36.8% of total 16S sequences), *Streptococcus* (21.6%), *Haemophilus* (12.6%), and *Corynebacterium* (11.3%). Each of these four genera also were present in >90% of samples. The biodiversity of the control group, estimated by Shannon’s Diversity index (H), decreased significantly over the course of the study (*p* = 0.02), but was unchanged in the PHiD-CV group (*p* = 0.85), as shown in Table 2.

**Fig 2:**
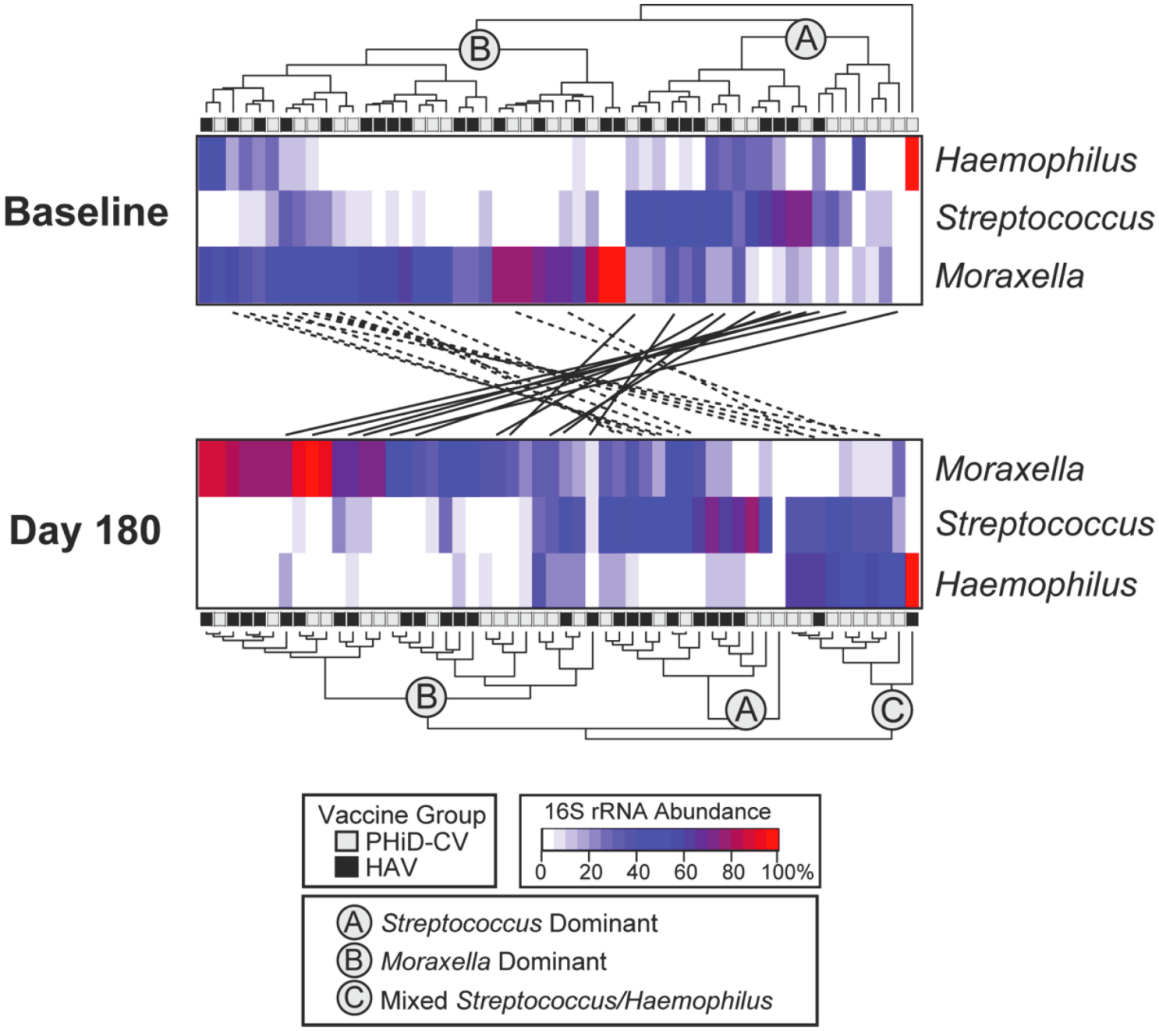
Clustering of subjects by abundances of *Haemophilus spp*., *Streptococcus spp*., and *Moraxella spp*. Subjects were grouped by hierarchical clustering on the basis of species-level percent 16S rRNA sequence abundances. Percent abundances are proportional to gray scaling. The upper heatmap presents data for baseline nasopharyngeal microbiomes and the lower heatmap presents data 180 days after vaccination. Subjects were classified into three basic groups on the basis of this clustering: A. *Streptococcus* dominant; B) *Moraxella* dominant; and C) Mixed *Streptococcus/Haemophilus* dominant. Solid lines connecting the two heatmaps indicate individuals that changed from cluster A to cluster B. Dotted lines indicate subjects that moved from cluster B to either cluster A or C. Black and gray boxes adjacent to dendogram designate vaccination group (PHiD-CV: 10-valent pneumococcal non-typeable *H*. *influenzae* protein-D conjugate vaccine; HAV: Hepatitis A vaccine).

Hierarchical clustering of subjects based on the genus-level compositions of their nasopharyngeal microbiomes revealed two prevalent bacterial communities (“nasopharyngeal-types”) at baseline, one dominated by *Streptococcus spp*. (43% mean sequence abundance; Cluster A, Fig 2) and the other by *Moraxella spp*. (54% mean sequence abundance; Cluster B, Fig 2). A third cluster consisting of a mixture of *Streptococcus spp*. (37% sequence abundance) and *Haemophilus spp*. (55% mean sequence abundance) was evident at the 180-day timepoint (Cluster C, Fig 2). Membership in a particular cluster was not associated with vaccination group or other demographic factors measured in this study, including age, sex, second-hand cigarette smoke exposure, and school attendance (data not shown).

Over the six-month study period, 28/54 (52%) of subjects switched from one nasopharyngeal-type to another, while the remaining 26/54 (48%) maintained their profile. Demographic comparisons showed that older age was significantly associated with profile shifts; (median age 24 months in the stable group vs. 44 months in the shifting group; *p* = 0.05). Other comparisons between stable and shifting communities yielded no significant associations (data not shown).

## Discussion

This study of primary vaccination with PHiD-CV among young Kenyan children found no changes in the prevalence or relative abundance of *S*. *pneumoniae* or *H*. *influenzae* following vaccination with either PHiD-CV or a control hepatitis A vaccine. Moreover, no increase in the abundance or prevalence of other potential pathogens, such as *M*. *catarrhalis* or *S*. *aureus*, was noted. Only the Shannon diversity index differentiated the control and PHiD-CV groups; whereas diversity decreased significantly in the control group between timepoints, no such changes were noted among subjects receiving PHiD-CV (Table 2). Overall, these results suggest that replacement of vaccine strains of *S*. *pneumoniae* by non-vaccine strains may have stabilized the nasopharyngeal microbiome against vaccine-mediated dysbiosis.[22]

The direct protective benefit of PHiD-CV arises from generation of antibodies that protect against invasive disease and against acquisition of nasopharyngeal carriage of vaccine serotypes. Reductions in vaccine-serotype carriage have been observed to occur within one-to-two months of vaccination with PHiD-CV.[6] Subsequent increases in carriage of non-vaccine serotypes occur over a longer period of time. Therefore, shifts in the overall nasopharyngeal microbiome may have occurred transiently after initial vaccination, but were undetectable at day 180, 4 months following the second dose. Biesbroek et al, assessed the nasopharyngeal microbiome in Dutch children following a 3-dose series of PCV-7 or control vaccine. The microbiota profiles of PCV-7 and control vaccine recipients differed significantly one month following completion of a 3-dose series but no differences between the two groups were detected 12 months later.[23]

We observed no change in the abundance or prevalence of *S*. *aureus* carriage following 2 doses of PHiD-CV. In contrast, an inverse relationship between carriage of *S*. *pneumoniae* and *S*. *aureus* was reported in cross-sectional studies conducted prior to PCV use and was also documented in one randomized-controlled trial of a 9-valent PCV.[5, 24, 25] Since PCV introduction, one population-level assessment in the Netherlands reported an increase in *S*. *aureus* carriage associated with PCV7 use; however, a follow-up study in the same population suggests it was a transient phenomenon.[26, 27] In addition, it’s important to note that *S*. *aureus* is best detected in the anterior nares but was cultured in this study, and the cited studies, from the posterior nasopharynx. Furthermore, the depth of 16S rRNA sequencing may have been inadequate to accurately measure the prevalence and abundance of a species such as *S*. *aureus*, which may be relatively sparse in the nasopharynx.

In this study, we observed co-carriage by *S*. *pneumoniae* and *H*. *influenza* in the majority of baseline samples. Moreover, the results of our exploratory statistical analysis (Fig 2, Cluster C) revealed several subjects in whom *S*. *pneumoniae* and *H*. *influenzae* co-existed at high abundances, relative to other members of the nasopharyngeal microbiome. The relationship between *S*. *pneumoniae* and *H*. *influenzae* in the nasopharynx was recently reviewed by Dunne *et al*.[28] The majority of studies suggest a positive association, although some have found the opposite. Recent microbiome studies have found that the relationship between *S*. *pneumoniae* and *H*. *influenzae* may be serotype dependent.[29] In our analysis, the abundances of *M*. *catarrhalis* and *S*. *pneumoniae* were negatively correlated (Pearson correlation coefficient = -0.28) and one or the other organism dominated the nasopharyngeal microbiomes of most subjects. On an individual level, patterns of microbiome composition were unstable over the course of this study (i.e., the dominant species often differed within an individual between baseline and the day 180 samples), indicating that the nasopharyngeal microbiomes among the study participants were in a state of flux. The host, environmental, and/or microbiological factors governing the dynamics in this ecosystem remain to be fully elucidated, but potentially could be exploited to modify the carriage of opportunistic pathogens that normally reside within mucosal reservoirs such as the airways and intestinal tract.

Although this research enhances our understanding of the effect of pneumococcal vaccination on the nasopharyngeal microbiome, several limitations must be addressed. First, we selected relatively few individuals (N = 60) for microbiome sequencing, thus limiting our ability to detect small differences in microbiome composition resulting from vaccination. For example, at the 180-day timepoint, we had 80% power to detect a difference in relative abundance between treatment groups of 0.1 for *Haemophilus influenzae*, whereas our observed mean difference was 0.015. Hence, this analysis was not powered to detect subtle differences between the two treatment groups. Second, the baseline specimen was collected in the dry season and the follow-up specimen was collected 180 days later during the rainy season; seasonal variation in *S*. *pneumoniae* and *H*. *influenzae* carriage may have confounded comparisons across time within the same group but this would not affect comparisons between groups.[30] Finally, the coarse phylogenetic resolution of the 16S gene precludes strain-level analysis, so changes in vaccine strain types were not detectable using this technology.

In conclusion, the nasopharyngeal microbiomes of Kenyan children are temporally variable but fall into “nasopharyngotypes” based on *Streptococcus*, *Moraxella* and *Haemophilus* abundance. Administration of two doses of PHiD-CV to children aged 12–59 months did not significantly alter the species composition or diversity of nasopharyngeal microbiomes in the study population. This suggests limited opportunity for replacement carriage with pathogens other than non-vaccine strains of *S*. *pneumoniae* or subsequent shifts in the microbiome related to such changes.

## Supporting Information

S1 Table Patterns of co-occurrence of *S*. *pneumoniae*, *H*. *influenzae*, and *M*. *catarrhalis* in nasopharyngeal specimens. (DOCX)
