# Supplementary material for: Effects of Vaccination with 10-Valent Pneumococcal Non-Typeable Haemophilus influenza Protein D Conjugate Vaccine (PHiD-CV) on the Nasopharyngeal Microbiome of Kenyan Toddlers
Source: PLoS One. 2015 Jun 17;10(6):e0128064. doi: 10.1371/journal.pone.0128064 (PMC4471099; doi:10.1371/journal.pone.0128064)
Supplement: S1 Table — (DOCX) [file pone.0128064.s001.docx]

**Supplemental Table 1**. Patterns of co-occurrence of *S. pneumoniae*, *H. influenzae,* and *M. catarrhalis* in nasopharyngeal specimens.^1^

|  |  | *S. pneumoniae* | | | | *H. influenzae* | |
| --- | --- | --- | --- | --- | --- | --- | --- |
|  |  | Pyrosequencing | | Culture | | Pyrosequencing | |
|  |  | **+** | **-** | **+** | **-** | **+** | **-** |
| *H. influenzae* | **+** | 32 | 6 | 27 | 8 | *n.a.* | *n.a.* |
|  | **-** | 10 | 6 | 8 | 11 | *n.a.* | *n.a.* |
| *M. catarrhalis* | **+** | 41 | 11 | *n.d.* | *n.d.* | 37 | 15 |
|  | **-** | 1 | 1 | *n.d.* | *n.d.* | 1 | 1 |

^1^ The baseline presence of each species was determined in all 54 study participants by either 16S rRNA pyrosequencing and/or culture. Cells present the number of individuals positive for one or both species indicated, as determined by each methodology.

*n.a.*: not applicable. *n.d.*: no data.
